# Preventing sickness absenteeism among employees with common mental disorders or stress-related symptoms at work: Design of a cluster randomized controlled trial of a problem-solving based intervention versus care-as-usual conducted at the Occupational Health Services

**DOI:** 10.1186/s12889-017-4329-1

**Published:** 2017-05-12

**Authors:** G. Bergström, M. Lohela-Karlsson, L. Kwak, L. Bodin, I. Jensen, M. Torgén, L. Nybergh

**Affiliations:** 1 Division of Intervention and Implementation Research in Worker Health, Institute of Environmental Medicine, Karolinska Institutet, SE-171 77 Stockholm, Sweden; 2 Centre for Occupational and Environmental Medicine, Stockholm County Council, SE-113 65 Stockholm, Sweden; 3 Department of Medical Science, Uppsala University, SE-751 85 Uppsala, Sweden

**Keywords:** Common mental disorders, Cluster randomized study, Stress-related disorders, Adjustment disorders, Depression, Exhaustion, Work environment, Occupational health services, Participative methodology, Problem solving therapy

## Abstract

**Background:**

Common mental disorders (CMDs) are among the leading causes of sick leave in Sweden and other OECD countries. They result in suffering for the individual and considerable financial costs for the employer and for society at large. The occupational health service (OHS) can offer interventions in which both the individual and the work situation are taken into account. The aim of this paper is to describe the design of a study evaluating the effectiveness of an intervention given at the OHS to employees with CMDs or stress-related symptoms at work. In addition, intervention fidelity and its relation to the outcome will be assessed in a process analysis.

**Methods:**

The study is designed as a cluster randomized trial in which the participating OHS consultants are randomized into either delivering the intervention or performing care as usual. Employees with CMDs or stress-related symptoms at work are recruited consecutively by the OHS consultants. The intervention aims to improve the match between the employee and the job situation. Interviews are held individually with the employee and the nearest supervisor, after which a joint meeting with both the employee and the supervisor takes place. A participatory approach is applied by which the supervisor and the employee are guided by the OHS consultant and encouraged to actively take part in problem solving concerning the work situation. Outcomes will be assessed at baseline and at six and 12 months. A long-term follow-up at 3 years will also be performed. The primary outcome is registered sickness absence during a 1-year period after study inclusion. Secondary outcomes are mental health and work ability. The intervention’s cost effectiveness, compared to treatment as usual, both for society and for the employer will be evaluated. A process evaluation by both the OHS consultants and the employee will be carried out.

**Discussion:**

The study includes analyses of the effectiveness of the intervention (clinical and economic) as well as an analysis of its implementation at the participating OHSs. Possible methodological challenges such as selection bias and risk of contamination between OHS consultants delivering the experimental condition and consultants giving usual care are discussed.

**Trial registration:**

ClinicalTrials NCT02563743 Sep 28 2015.

## Background

Common mental disorders (CMDs) usually include depression, anxiety and adjustment disorders. They are the leading cause of all sick leave lasting for more than 14 days in Sweden [1] as well as being highly prevalent in several other OECD countries [2]. In Sweden, 50 % of all newly granted disability benefits awarded to women in December 2015 were due to mental health problems; the figure for men was 37% [1].

It has been estimated that the cost of mental health problems amounts to approximately €136 billion per year in Europe [3]. For the afflicted individual, mental health problems give rise to considerable suffering and, in the long run, an increased risk of social isolation and a substantial deterioration in personal finances. Furthermore, people on long-term sick leave for mental health problems have an increased risk of mortality from suicide or diseases such as cardiovascular diseases and cancer [4].

Recent systematic reviews have found that work-environment factors such as high job demands combined with low levels of control at work increase the risk of mental health problems [5, 6]. Other work-environment factors that heighten the risk of CMDs include low social support, job insecurity and an imbalance between work effort and the reward or recognition that the work receives. The risk of future mental health problems is lower among employees who believe that they are treated fairly at work and are able to influence their work [5]. Several calls for further intervention research within this area have been made [7, 8].

Interventions aimed at preventing or reducing work-related mental health problems can be conducted at the organizational, work group or individual level or combinations of these levels. Interventions aiming at return to work (RTW) or the reduction of sick leave evaluated in randomized controlled trials (RCTs) are often conducted at the individual level without actively including the workplace [9]. Individuals on sick leave due to CMDs, and for whom a change in their work situation is important, may hence risk new periods of sick leave and continued mental health problems if the workplace is not involved. Indeed, recent research suggests that adding a workplace-focused approach to a clinical intervention is effective in improving work return among employees suffering from depression [10]. Furthermore, studies evaluating workplace-focused interventions for mental health problems have found some support for an improvement in RTW among employees receiving problem-solving therapy (PST) [11, 12] or cognitive behavioural therapy (CBT) [13].

Interventions for improving RTW, such as PST or workplace-focused measures aimed at improving workability among employees [14, 15] may also be effective in preventing sickness absenteeism among employees with mild mental problems or occupational stress who continue to work but are at risk of future sick leave. A study by Lexis et al. [16] evaluated an intervention based on CBT and PST techniques among bank personnel who were not on sick leave but had mild to severe depression. The control group received care as usual consisting of a consultation with an occupational physician. The results showed a statistically significant lower total amount of sickness absenteeism, and a lower proportion of depressive symptoms among the intervention group compared to the control group after 12 months. Other published studies of early interventions among employees at risk of future mental health problems, reduced work functioning and/or sick leave have produced varying results (e.g [17–21]).

The occupational health service (OHS) has knowledge of the employee’s work environment and can offer interventions to prevent sickness absenteeism which take both the individual and the work situation into account. To the best of our knowledge, this is the first RCT of an intervention for employees with stress-related symptoms or CMDs conducted in a Swedish OHS setting.

The aim of this paper is to describe the design of a study which evaluates an individually- and workplace-focused intervention given at the OHS to employees with CMDs or stress-related symptoms at work. In particular, the intervention’s impact on sick leave and mental health as well as its cost-effectiveness will be studied. Furthermore, a process evaluation will be conducted to assess intervention fidelity and its association with future sick leave.

## Methods

### Study design and setting

**Fig. 1:**
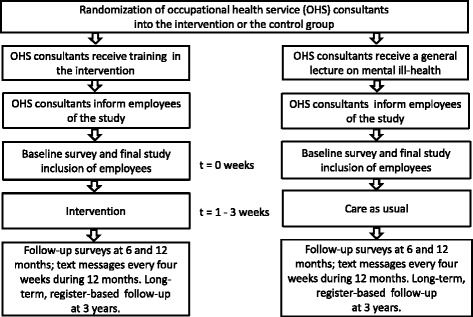
Study design and overview of the trial

The study has a two-armed cluster randomized controlled design with a primary follow-up period of 1 year (see Fig. 1). The study is conducted in collaboration with three OHSs in Sweden. One OHS has nationwide coverage while two are regional OHSs. Each participating OHS consultant is considered as one cluster. The participating OHS consultants consist of occupational nurses, physicians, physiotherapists, ergonomists, occupational health counselors and psychologists.

### Ethical approval

Ethical approval has been granted by the Ethical Review Board in Stockholm (registration number 2015/549–31/1). The study complies with the ethical guidelines regarding voluntary participation, privacy and the handling of personal data in accordance with Sweden’s Personal Data Act and Secrecy Act. Informed consent will be obtained from all participants and will include, amongst other things, information about the right to opt-out from the study at any time without giving a reason and without any consequence for the help received from the OHS (see also Recruitment of participants).

### Recruitment of participants

The OHS consultants provide preliminary information about the study to employees seeking care at the OHS for work-related stress symptoms or CMDs which fulfil the inclusion criteria. They then ask whether the employee’s contact information may be sent to the research group at Karolinska Institutet (KI). Written consent to transfer this information to KI is obtained from those who are preliminarily interested in participating in the study. Subsequently, members of the research group contact the employee and give both verbal and written information (sent by email) about the study and what participation would entail. Final consent is obtained and study inclusion achieved when the employee has submitted the baseline questionnaire and confirmed having taken part of the information about the study by ticking a box at the end of the baseline questionnaire. Paper-and-pencil questionnaires are offered to those who prefer this option. Recruitment of participants began in September 2015 and will be completed during the spring 2017.

### Inclusion criteria

Inclusion criteria are met by employees fulfilling the following criteria:

- The employee seeks help at the OHS for occupational stress affecting their ability to work. If the employee is on sick leave due to adjustment disorders, anxiety or depression, the period of sick leave should not have exceeded 3 months at the time of the first OHS visit.
- The employee should accept involvement of the supervisor in the intervention.
- The employee also needs to understand both written and spoken Swedish as the study questionnaires and text messages are in Swedish.

### Exclusion criteria

Employees will be excluded from the study if they are pregnant at the time of inclusion, victims of workplace bullying, have post-traumatic stress-disorder, severe mental illness (e.g. psychosis) or any other co-morbidity that could considerably affect their ability to work and/or quality of life.

### Randomization

The study uses a two-armed cluster randomized design in which the participating consultants are randomized using computer-generated random numbers into either giving the intervention or performing care as usual. Hence, each OHS consultant has to continuously apply the same treatment within the context of this study. The randomization is stratified by OHS unit as well as by occupational category, as those two factors are assumed to have a potential impact on the results and a balance within strata is necessary.

It was not feasible to randomize employees between OHS consultants trained or not trained in the intervention, because different employers have contracts with different OHS consultants, that is, a certain employee is linked to a certain OHS consultant. An alternative may have been to train all OHS consultants in the intervention, however, this should have increased the risk of contamination.

### Blinding

The employees are not given information about the possibility of receiving another type of treatment within the trial. This is because the OHS consultants have been pre-randomized into delivering one of the two interventions. The researchers will not know about treatment allocation during the analyses. However, the OHS consultants are not blinded with regard to which treatment is given.

### Matched control group

An additional comparison group for those on sick leave at baseline will be obtained by means of register-based data from the SSIA. The material will be matched on the basis of diagnosis, age, sex and sick leave during the previous 3 months. This data allows for a better understanding of the development patterns of sick leave for the study participants on sick leave in both the control and the intervention groups.

### The individual and workplace-focused intervention

The OHS consultants delivering the experimental condition receive general information about the study and information about the recruitment process. After this, a 1-day training course is given by members of the research group and a clinical psychologist. Individual supervision and follow-up is thereafter provided on a continuous basis by the KI research group by email and scheduled phone calls. The OHS consultants are also offered further guidance for the intervention at 1–2 follow-up meetings after the initial training (on demand). They also receive detailed work sheets.

The theoretical basis for the intervention stems from PST [22] and the “mismatch” model concerning the match between the employee and the work environment [23]. This mismatch model emphasizes six aspects of the work situation which are addressed during the meetings: workload, control, reward, community, fairness and values. A participatory approach is applied by which the supervisor and the employee are guided by the OHS consultant and encouraged to actively take part in problem solving concerning the work situation [14, 24].

**Fig. 2:**
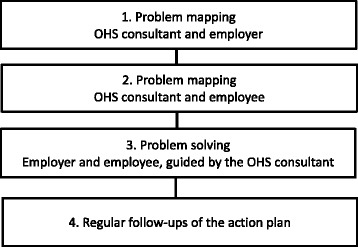
Overview of the intervention

As outlined in Fig. 2, the intervention comprises two interviews, one of which is held with the employer (usually the nearest supervisor) and the other with the employee, and a third session at which both the employer and the employee are present [14, 24]. This third dialogue aims to facilitate a closer match between the employee’s expectations and abilities and the job situation. The order in which the two separate interviews are held depends on the internal logistics at the OHS.

The interview with the employee’s supervisor covers any earlier measures taken by the employer for the employee and the supervisor’s view of the “causes” of the employee’s stress or poor mental health. The supervisor is also asked about what he/she considers to be the most important problem to address to promote the employee’s work ability or RTW. For practical reasons this meeting usually takes place over the phone and lasts about 15 to 30 min. The same themes are discussed during the interview which takes place in person between the OHS consultant and the employee. However, this session is more comprehensive and also includes an initial section concerning the private situation of the employee, work-family balance, and a survey of possible mismatches between the employee and the job situation [23]. A problem-solving approach is also initiated. This interview takes approximately 90 min.

The problem-solving approach is guided by the following steps (1) goal setting and problem definition – the problem is specified in detail including when and where it happens as well as how it is expressed, (2) generation of possible solutions, (3) decision making about solutions, including potential consequences of different solutions, (4) setting up an action plan, including implementation of prioritized solutions.

During a third meeting the supervisor, employee and the OHS consultant meet face-to-face and focus on problem definition and possible solutions for promoting work ability or RTW. At this meeting the supervisor’s and the employee’s views of the problem are discussed with the aim of achieving a convergence of views. Relevant external parties such as an OHS physician or a representative of the Social Insurance Agency may also participate in this meeting if deemed necessary. The main task of the OHS consultant during the third meeting is to help the employee and his/her supervisor to reach specific problem definitions and feasible solution(s) to be implemented at the workplace to improve work ability or facilitate RTW. The suggested solutions and their follow-up are registered in an action plan by the OHS consultant. It is crucial that both the employee and the supervisor are encouraged to understand, and to be active and participatory in, the problem-solving process and that consensus between the parties is reached on possible solutions to facilitate RTW or manage stress at the workplace.

The OHS consultant is recommended to conduct regular follow-ups of the supervisor and the employee by phone calls or meetings in accordance with an agreed schedule. At least three follow-ups during the 3 months following the third session are recommended. These follow-ups focus on the action plan and whether planned measures have been undertaken; progress and possible new problem solving. In addition to the planned intervention, the OHS may also refer the employee to other services or measures in accordance with routine practice.

### Care as usual

The OHS consultants delivering the control condition receive information about the recruitment process followed by a general introduction to research into psychosocial factors and mental health at work. In total this takes 2 h.

The OHS consultants then continue to deliver the routine care throughout the study period. At the three OHSs where the current study is being performed, care as usual in case of stress-related symptoms and CMDs usually involves both the employee and the supervisor. However, these practices do not follow the same structure as in the experimental intervention nor do they use the mismatch model as guidance for defining problem areas or the described problem-solving approach. At one of the OHSs the intervention is more focused on further medical and lifestyle factors, even though the work situation is also addressed and the employer is involved in the intervention. There is also some variance among the OHS consultants as to how this intervention is applied. One of the OHSs does not usually hold an individual interview with the employee; in other words, the joint meeting between the employee, the employer and the OHS consultant is often held directly after a short interview with the supervisor. A third OHS is about to implement the Swedish guidelines for OHSs concerning the management of mental health at work. These guidelines recommend the involvement of the employer and problem-solving techniques. However, despite the similarities with the experimental intervention the personnel delivering the care as usual have not received the same training in these methods as the consultants delivering the active intervention. A survey of the OHS consultants regarding more specific details of the contents and routines of care as usual will also be conducted as part of the study’s process analysis.

### Co-interventions

As it is difficult to avoid the possibility of co-interventions, the questionnaires sent out to the employees ask for information about supplementary treatment from the OHS or other agencies. The questionnaire assesses the type of intervention or help that was sought as well as the number of times it was received and whether it was administered by the OHS or others.

### Measurements and procedure

Data are gathered by use of registers, questionnaires and SMS text messages. Register data are collected from the SSIA. These data are based on register information, including sick-listing and sickness and activity compensation. Valid questionnaires in electronic format are administered at baseline and after six and 12 months (paper versions are available on demand). Furthermore, monthly (every fourth week) text messages asking five questions about sick leave, stress and work ability obtained from the employee are sent to the participants over a 1-year period [25]. The use of text-messages is motivated since this measurement also includes short spells of sick leave (<15 days) which are not covered by the SSIA. A 3-year follow-up of register-based data on sick leave is also planned.

### Primary outcome

#### Registered sickness absenteeism

The primary outcome is the total number of days of sickness absence during the 12-month follow-up period (including sickness benefit and disability pension). Sickness benefit and disability pension will also be analyzed separately.

### Secondary outcomes

#### Self-reported sickness absence and return to work using text messages

For those on sick leave at baseline, the time to RTW will be calculated from baseline until (1) the employee returns to work in any increased level, or (2) full return to work (working usual hours during an uninterrupted period of at least 4 weeks). The prevalence of no sick leave, part-time sick leave and full-time sick leave during the 12-month follow-up period following baseline will be presented.

#### Registered sickness absenteeism

The total number of days of registered sickness absence from the SSIA will be calculated in the same way after 3 years as at the 1-year follow-up.

#### Mental health and stress-related symptoms

Self-reported exhaustion will be assessed by the Emotional exhaustion sub-scale of the Maslach Burnout Inventory - General Survey (MBI-GS). The MBI-GS is a well-established instrument for assessing burnout [26]. Furthermore, stress-related Exhaustion Disorder is assessed by the s-ED measurement instrument [27]. The s-ED is based on diagnostic criteria for stress-related Exhaustion Disorder according to the Swedish National Board of Health and Welfare. Support has been found for its constructive and predictive validity [27]. In addition, the Hospital Anxiety and Depression Scale will be used to assess anxiety and depression among the employees [28, 29]. Finally, self-reported stress as assessed by a single item included in the text messages will be used. This has been found to be valid for monitoring stress in a work-related context [30].

#### Health problems

Sleeping problems will be measured by the insomnia subscale from the Karolinska Sleep Questionnaire [31]. Furthermore, the European Quality of Life - 5 Dimensions questionnaire (EQ-5D) will be applied to investigate health-related quality of life [32]. Self-perceived general health will be assessed by a single question (Orwelius et al. In manuscript). Presenteeism (being at work while sick) will be assessed by a single question developed by Aronsson et al. [33].

#### Work ability and working environment

Self-reported work ability will be assessed by three items from the Work Ability Index [34]. Two of the items relate to perceived capacity to work relative to the physical and mental demands of the work. The third item enquires whether the employee believes that s/he can be working at the same workplace in 2 years’ time. Two additional items are intended to measure work performance impairment due to (1) health problems and (2) problems in the working environment. These items have been developed and modified inspired by the Work Productivity Activity Impairment questionnaire - General Health Questionnaire (WPAI-GH) [35, 36]. The second item has been developed and evaluated in Sweden [35–37]. Finally, job satisfaction will be estimated by a single self-report item [38].

#### Prognostic measures

The demand-control-support model [39] will be considered as a possible prognostic measure in this study. The study will also include items from the General Nordic Questionnaire (QPSNordic) relating to ongoing conflicts with the superior, perceived loss of control over work tasks and conflicts between the employee’s values and how the work is actually carried out [40]. One self-efficacy item about how confident participants are about whether they will be back to their usual working hours in their usual work position 3 months after the baseline measurement will be developed and validated within the project. Finally, the OHS consultants will be asked a similar question about whether the employee will be back to his/her usual working hours in 3 months’ time.

### Cost-effectiveness

A full economic evaluation of costs for society and for the employer will be conducted [41]. The expenses that will be collected are direct costs, such as intervention costs paid by the employer and possible rehabilitation provided by other caregivers. Indirect costs such as time used by participants for the intervention and travel time will also be collected. Time for the intervention will be calculated on the basis of the intervention program. Time for travel to attend the meetings will be standardized at 1 h per meeting (30 min/single way). Treatment costs will be estimated using national unit costs and the fee charged by the OHS. Lost time due to treatment or travel will be evaluated using national median wages. Information about absenteeism and reduced performance due to health-related and work environment-related problems, i.e. productivity loss, will be collected using validated questions and used to calculate potential benefits of the intervention. Productivity loss will be estimated using national median wages.

Data regarding the direct costs and potential savings which emerge from the intervention will be collected in parallel with the implementation of the project and for a 1-year follow-up period. All costs and consequences will be converted to a single year using the consumer price index. Discount of costs or consequences is not needed for the 1 year follow-up because of the short follow-up period. Costs or savings that occur during the long term follow-up will be discounted using a 3 % discount rate [42]. The employer’s economic evaluation will be conducted using a cost benefit analysis; the evaluation of the economic costs for society will be conducted using a cost effectiveness analysis.

### Process evaluation

The importance of a process evaluation for studies conducted within the OHS has been highlighted in previous research [43]. The current study will perform a process evaluation of both the OHS and the employee [44]. The purpose of this is to examine: the attitudes of the OHS consultants towards the study; the organizational support for the study (on the part of the OHS-consultants); the content of the control condition and the fidelity of the OHS-consultants to the experimental condition.

The participating OHS consultants´ attitudes towards and knowledge of the research project will be evaluated using a short questionnaire at the start of the study. Examples of items are “To what degree do you feel it is good to be part of the project?” or “To what degree do you understand the aim of the project?”

The OHS consultants in the experimental condition will be asked to rate their overall fidelity to the method for each employee during the study (this is done directly after the joint meeting with the supervisor and the employee). Telephone follow-ups with each participating consultant are conducted (both for the experimental and the control group) during the recruitment (and intervention) process. These follow-ups include questions about recruitment, such as clarifications of the inclusion criteria, as well as logistical or contextual factors related to recruitment. For the consultants delivering the experimental condition, issues concerning the feasibility of the intervention and the fidelity of the OHS consultant are examined. The meetings are summarized in short notes.

After the recruitment period is finished, fidelity to the intervention among the OHS consultants delivering the experimental intervention will be assessed by means of check-lists that will be built around key aspects of the intervention. Examples of such variables are whether contact with the workplace has been maintained and whether employees have been active in the problem-solving process and the planning of future measures. The content and fulfillment of the recommended follow-ups with employees and supervisors will also be addressed. The OHS consultants in the control condition will be given a check list where they are asked to describe the content of the characteristics of the control condition (intervention).

For all OHS consultants in the experimental and the control condition, the check lists will also include questions about the recruitment process (for instance, whether all employees who should be informed about the study actually received this information). Furthermore, organizational-level variables will be addressed, such as how the research project was introduced to the OHS consultants by the management at the OHS and how the study was supported while it was in operation.

Employee satisfaction with the intervention or treatment as usual will be assessed 6 months after the completed intervention. The assessment will consider aspects such as the quality of communication with OHS personnel, the relevance of the intervention, problem-solving skills learned during the intervention, planned adjustments at work, influence on the planned adjustments, agreement with the supervisor, the implementation of planned adjustments, whether follow-up contacts with the OHS were held, and questions about treatment satisfaction.

### Data analysis

Statistical methods adapted for cluster randomized designs will be applied. The plan is to apply various forms of regression models and mixed models. Intention-to-treat analyses will be carried out. Where relevant, per-protocol analyses will also be performed. If it appears that potential confounders have been unevenly distributed and can be expected to affect the results when the two interventions are compared, these factors will be adjusted for in the analyses. Possible interaction effects on the outcome for (1) gender x treatment (intervention) and (2) sick leave status (sick leave/no sick leave) x treatment will be checked for. If they are statistically significant, subgroup analyses will be considered.

#### Statistical power

Sample sizes of 75 in Group 1 and 75 in Group 2, which were obtained by sampling 15 clusters with an average of five subjects each in Group 1 and 15 clusters with an average of five subjects each in Group 2, achieve 84% power to detect a difference between the group means of at least 25.00 days of registered sickness absence 1 year after baseline. The standard deviation of subjects is 50.00. The intra-cluster correlation coefficient is 0.010. The coefficient of variation of cluster sizes is 0.650. A two-sided t-test was used with a significance level of 0.050. This test used degrees of freedom based on the number of subjects. No missing values are expected since the primary outcome is based on register data. The aim of the current study is therefore to include a total of 150 participants.

## Discussion

This study protocol presents the design of a cluster RCT performed at the OHS. The OHS is acquainted with the employee’s work environment and can offer measures that take both the individual and the working situation into account. The purpose of the evaluated intervention is to prevent sickness absenteeism and improve work ability among employees with CMDs or stress-related symptoms. The intervention’s impact on sick leave and mental health and its cost-effectiveness will be evaluated. By conducting a process evaluation of the intervention we also intend to evaluate how the treatment fidelity of the OHS-consultants may influence sickness absence outcomes among the participating employees. The process evaluation will also give information about the feasibility of the intervention and contextual factors that may have affected the implementation process, which could be useful for future applications of the intervention.

### Methodological considerations

We cannot exclude the risk of selection bias i.e. that the OHS consultants choose which employees to invite to participate in the study on the basis of, for example, how well they believe the intervention would work for a particular employee. Hence, during the training and follow-ups with the OHS consultants, we emphasize the importance of telling all employees who match the inclusion criteria about the study. We will also include an item relating to this in the process evaluation check-lists that will be distributed to the OHS consultants.

There is also risk of possible contamination of interventions because OHS consultants at the same OHS may deliver either care as usual or the experimental intervention. This concern is, similarly to the above, addressed in the training and follow-ups of the OHS consultants belonging to the intervention group, at which the reasons for not discussing the intervention contents with colleagues allocated to deliver the control condition are explained. An item on this issue will also be included in the process evaluation check-list to the OHS consultants.

In Sweden, the employer records and pays for sick leave related costs up to and including the 14th day of sick leave. For this reason, national, register-based data on sick leave maintained by the SSIA is available from the 15th day onwards, with no information available for short-term sick leave. The current study consequently sends text messages that include questions about short-term sick leave (< 15 days) to all participants once every 4 weeks. This will give valuable information about short-term sick leave among the participants. Nevertheless, the reliance on similar self-reports is subject to possible recall bias and larger drop-out rates than register-based data. To increase the response rates, we have used reminders by calling employees about missing answers; in a previous study this was found to increase response rates [45].

Two previous Swedish studies have shown that employees with CMDs may seek treatment on their own, especially if they are aware that they belong to the control group [46, 47]. To detect possible treatment bias of this sort, items on co-interventions have been included in the questionnaires to the employees. The OHS consultants have also been advised not to inform employees whether they belong to the control or the intervention group, since active workplace-focused treatments are given in both conditions.

The study population will consist of employees with reported stress-related symptoms but no sick leave at baseline and employees on sick leave due to CMDs. As mentioned earlier, possible differential effects of the intervention in these groups may arise. Potential interactions between sick leave status at baseline and the secondary outcomes will therefore be taken into account.

### Possible impact of results

For employees with mental health problems or stress-related symptoms, failure to take the work environment into account may lead to reduced work ability and repeated and/or prolonged spells of sick leave. The current intervention looks at both the individual and the workplace context. If the intervention proves successful and is implemented at large within the OHS sector, it may result in increased work ability, reduced rates of sick leave and improved quality of life among employees with CMDs or occupational stress. This may also reduce costs, both for the employer and for society at large.

## Abbreviations

CBT: Cognitive behavioral therapy
CMD: Common mental disorders
MBI-GS: Maslach Burnout Inventory - General Survey
OHS: Occupational health services
PST: Problem solving therapy
RTW: Return to work
s-ED: The stress-related Exhaustion Disorder measurement instrument
SSIA: The Swedish Social Insurance Agency
